# Human metapneumovirus infection of organoid-derived human bronchial epithelium represents cell tropism and cytopathology as observed in *in vivo* models

**DOI:** 10.1128/msphere.00743-23

**Published:** 2024-01-24

**Authors:** Pau Ribó-Molina, Stefan van Nieuwkoop, Anna Z. Mykytyn, Peter van Run, Mart M. Lamers, Bart L. Haagmans, Ron A. M. Fouchier, Bernadette G. van den Hoogen

**Affiliations:** 1 Department of Viroscience, Erasmus Medical Center, Rotterdam, the Netherlands; University of Michigan, Ann Arbor, Michigan, USA

**Keywords:** human metapneumovirus, organoids, primary cell cultures, tropism, cytopathology

## Abstract

Human metapneumovirus (HMPV), a member of the *Pneumoviridae* family, causes upper and lower respiratory tract infections in humans. *In vitro* studies with HMPV have mostly been performed in monolayers of undifferentiated epithelial cells. *In vivo* studies in cynomolgus macaques and cotton rats have shown that ciliated epithelial cells are the main target of HMPV infection, but these observations cannot be studied in monolayer systems. Here, we established an organoid-derived bronchial culture model that allows physiologically relevant studies on HMPV. Inoculation with multiple prototype HMPV viruses and recent clinical virus isolates led to differences in replication among HMPV isolates. Prolific HMPV replication in this model caused damage to the ciliary layer, including cilia loss at advanced stages post-infection. These cytopathic effects correlated with those observed in previous *in vivo* studies with cynomolgus macaques. The assessment of the innate immune responses in three donors upon HMPV and RSV inoculation highlighted the importance of incorporating multiple donors to account for donor-dependent variation. In conclusion, these data indicate that the organoid-derived bronchial cell culture model resembles *in vivo* findings and is therefore a suitable and robust model for future HMPV studies.

**IMPORTANCE:**

Human metapneumovirus (HMPV) is one of the leading causative agents of respiratory disease in humans, with no treatment or vaccine available yet. The use of primary epithelial cultures that recapitulate the tissue morphology and biochemistry of the human airways could aid in defining more relevant targets to prevent HMPV infection. For this purpose, this study established the first primary organoid-derived bronchial culture model suitable for a broad range of HMPV isolates. These bronchial cultures were assessed for HMPV replication, cellular tropism, cytopathology, and innate immune responses, where the observations were linked to previous *in vivo* studies with HMPV. This study exposed an important gap in the HMPV field since extensively cell-passaged prototype HMPV B viruses did not replicate in the bronchial cultures, underpinning the need to use recently isolated viruses with a controlled passage history. These results were reproducible in three different donors, supporting this model to be suitable to study HMPV infection.

## INTRODUCTION

The human metapneumovirus (HMPV) was identified in 2001 in the Netherlands (1) and is a member of the *Pneumoviridae* family (2). HMPV infections can occur in people of all ages, but children of less than 5 years of age, the elderly, and immunocompromised patients are most susceptible to severe disease (1, 3–7). HMPV can be categorized in genotypes A and B, which were initially split into four sublineages: A1, A2, B1, and B2 (8). Viruses from the A1 sublineage have not been detected since 2006, while viruses from the other sublineages have continued to circulate and evolve, resulting in the emergence of newly described sublineages (9). For instance, in recent years, the circulation of viruses with a duplication in the attachment gene (G) of either 111 or 180 nucleotides (nt) was reported. These viruses all belonged to the newly defined lineage A2.2.2 (9–13).

Previously, experimental infections of cynomolgus macaques with HMPV NL/1/00 (A1) demonstrated that HMPV caused lesions throughout the respiratory tract, with ciliated epithelial cells as the main target of HMPV infection (14). Similarly, studies in cotton rats demonstrated the presence of viral antigens primarily on the apical side of epithelial cells throughout the respiratory tract (15). Despite these findings, *in vitro* studies with HMPV have been performed mostly in monolayer undifferentiated cells, which, in general, have a basal cell-like phenotype, which can present limitations when studying respiratory virus infections (as reviewed in reference 16). A limited number of studies have assessed HMPV replication in three-dimensional primary epithelial models, often with moderate success due to limited viral replication (17–21).

In this study, we established a differentiated primary cell culture model to study HMPV infection. Stem cells were isolated from adult bronchial tissue and were expanded into undifferentiated organoids. Subsequently, differentiation was performed at the air-liquid interface (ALI) generating cultures containing ciliated, club, goblet, and basal cells (22). This model has previously been demonstrated to be successful in studies with other respiratory viruses such as respiratory syncytial virus (RSV), parainfluenza virus 3, and severe acute respiratory syndrome-coronavirus-2 (23–25). Here, we demonstrate the sensitivity of the bronchial cultures derived from three different donors to a range of HMPV isolates. The described cellular tropism, induced cytopathic effects, and cytokine responses confirm the suitability of this model as a physiologically relevant and robust model for studying HMPV infection that closely resembles *in vivo* observations.

## RESULTS

### HMPV replicates in an organoid-derived bronchial culture model

**Fig 1:**
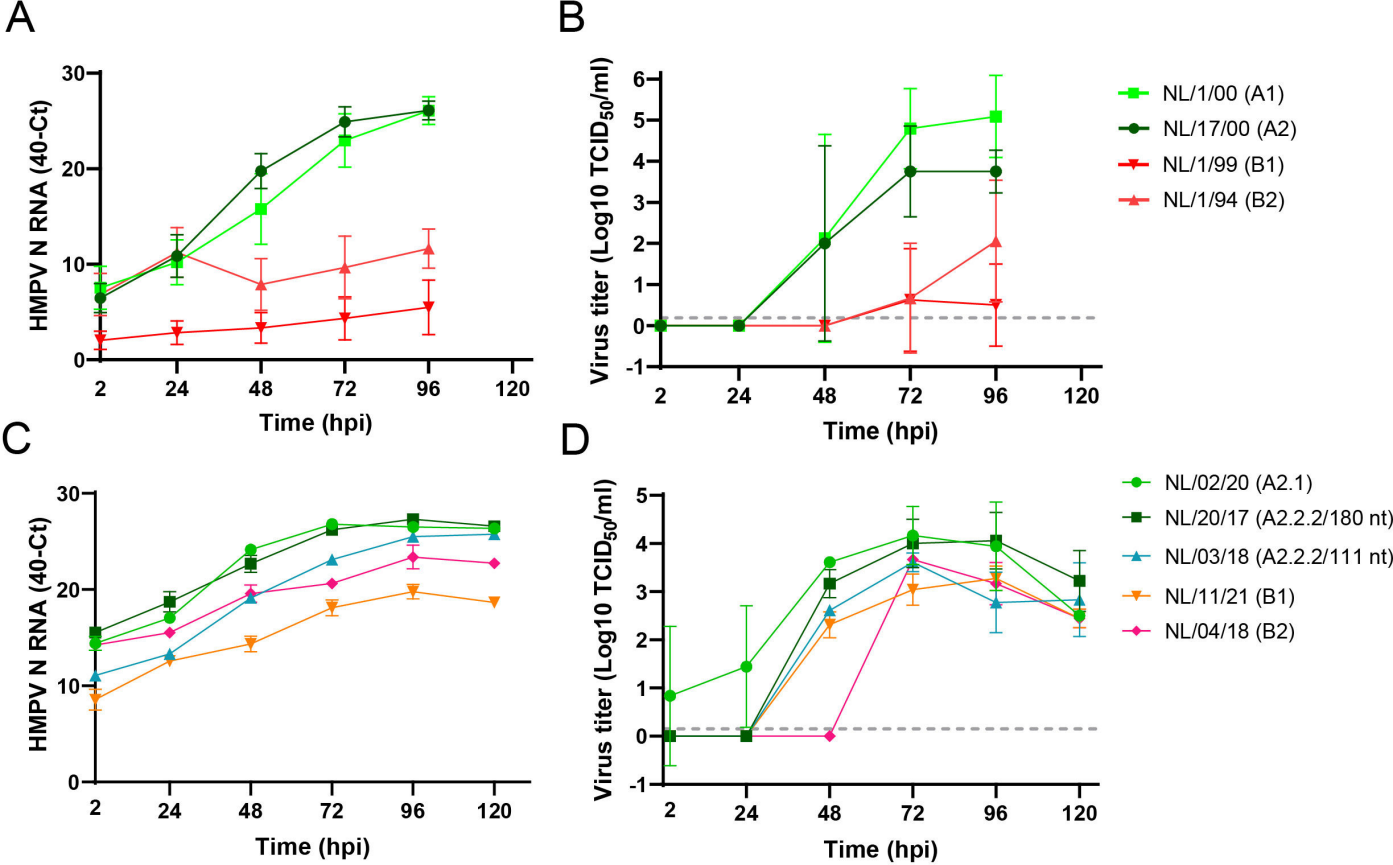
Replication kinetics upon inoculation of organoid-derived bronchial cultures at an MOI of 0.1 with HMPV prototype viruses of lineage A1, A2, B1, and B2 (A and B) or recent virus isolates from the different lineages (C and D). Replication was determined by measuring the increase of viral genomes with qRT-PCR assays (A and C) or the increase of viral titers with end-point titrations in Vero-118 cells (B and D). Time is expressed as hpi. The experiments were performed in triplicate. RNA levels are shown as mean with standard error of the mean. Viral titers are shown as geometric mean with standard deviation, and the limit of detection is shown with a gray dotted line at 1.5 TCID_50_/mL. Representative experiments in one donor and done in triplicate are shown.

**Fig 2:**
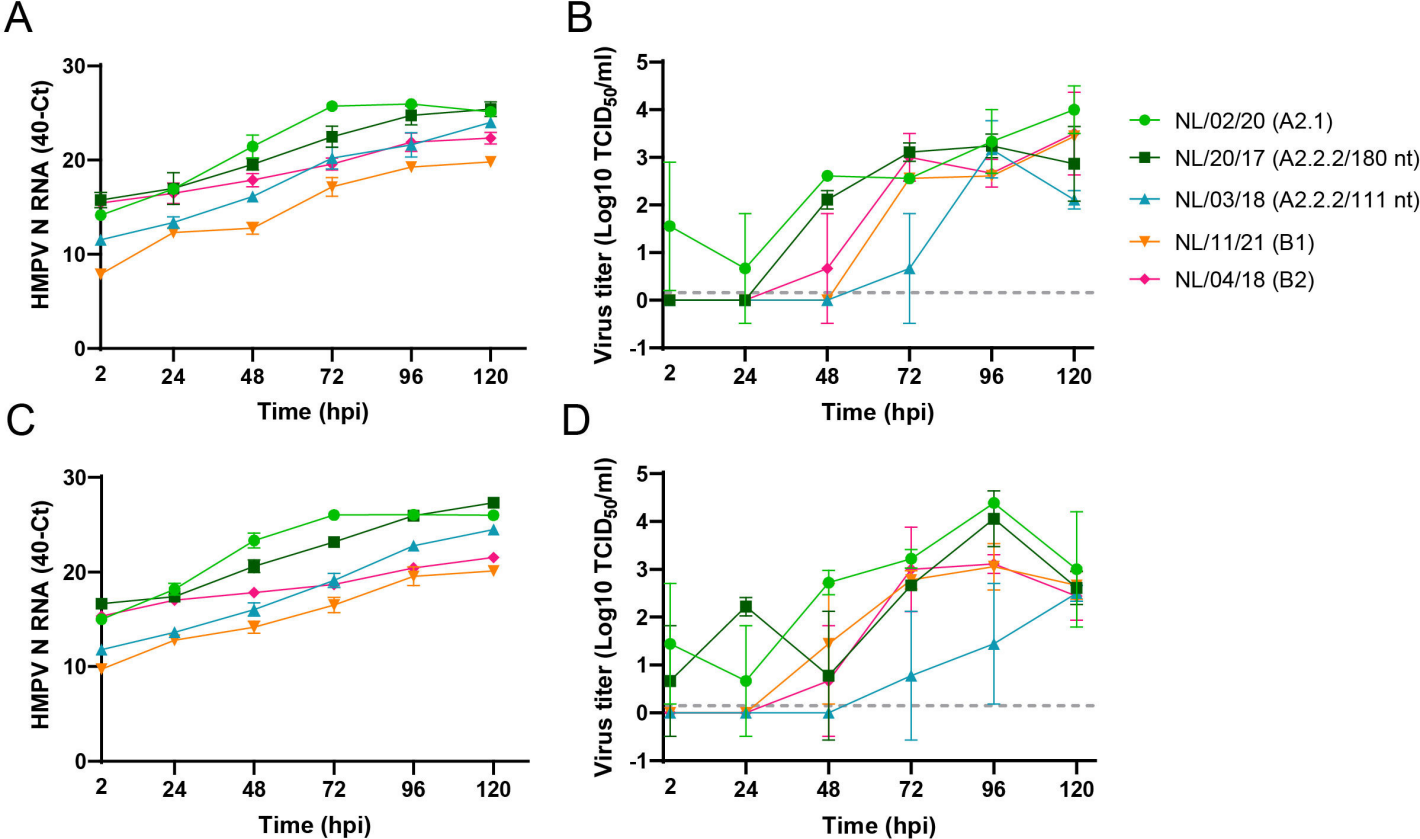
Replication kinetics of recent HMPV isolates upon inoculation at an MOI of 0.1 of organoid-derived bronchial cultures from two additional donors (panels A and B and panels C and D). Replication was determined as the increase of viral genomes and viral titers, as detailed in Fig. 1.

To assess the susceptibility of the organoid-derived bronchial culture model to HMPV infection, the cultures were inoculated with prototype viruses of the early defined four HMPV sublineages, NL/1/00 (A1), NL/17/00 (A2), NL/1/99 (B1), and NL/1/94 (B2). Replication kinetics were evaluated upon apical inoculation at an MOI of 0.1 and sampling of apical washes every 24 hours up to 96 hours post-inoculation (hpi). HMPV belonging to lineage A replicated more efficiently in the bronchial cultures than viruses from lineage B, as observed in terms of increase in viral RNA (Fig. 1A) and infectious virus particles (Fig. 1B). No major differences were observed between viruses from the same lineage (A or B). To examine whether the lower ability of HMPV genotype B viruses to replicate in the cultures was isolate or genotype specific, a set of recent genotype B virus isolates were examined in parallel with recent genotype A isolates, including isolates from the A2.2.2 lineage with either a 180 or 111 nt duplication in the G gene: NL/02/20 (A2.1), NL/20/17 (A2.2.2/180 nt), NL/03/18 (A2.2.2/111 nt), NL/11/21 (B1), and NL/04/18 (B2) (9). Upon inoculation of the organoid-derived bronchial cultures with these recent HMPV isolates at an MOI of 0.1, all viruses replicated. Here, HMPV isolates from the B lineages and the A2.2.2 isolate with the 111-nt duplication replicated to slightly lower levels than the HMPV A2.2.2/180 nt and HMPV A2.1 isolates, as detected by an increase in RNA levels (Fig. 1C), as well as in virus titers (Fig. 1D). These findings were reproduced in organoid-derived bronchial cultures from two other donors, which showed the robustness of the system (Fig. 2A through D). Replication kinetics with the recent HMPV isolates were sampled for an additional day compared to the replication kinetics studies for the prototype viruses, as it seemed that the replication of the recent isolates was slower than the prototype viruses in these cultures. The lower titers observed for the recent isolates could also be due to a less efficient infection of Vero-118 cells by the recent HMPV viruses than by the older prototype isolates, during titration.

### HMPV primarily infects ciliated cells in organoid-derived bronchial cultures

**Fig 3:**
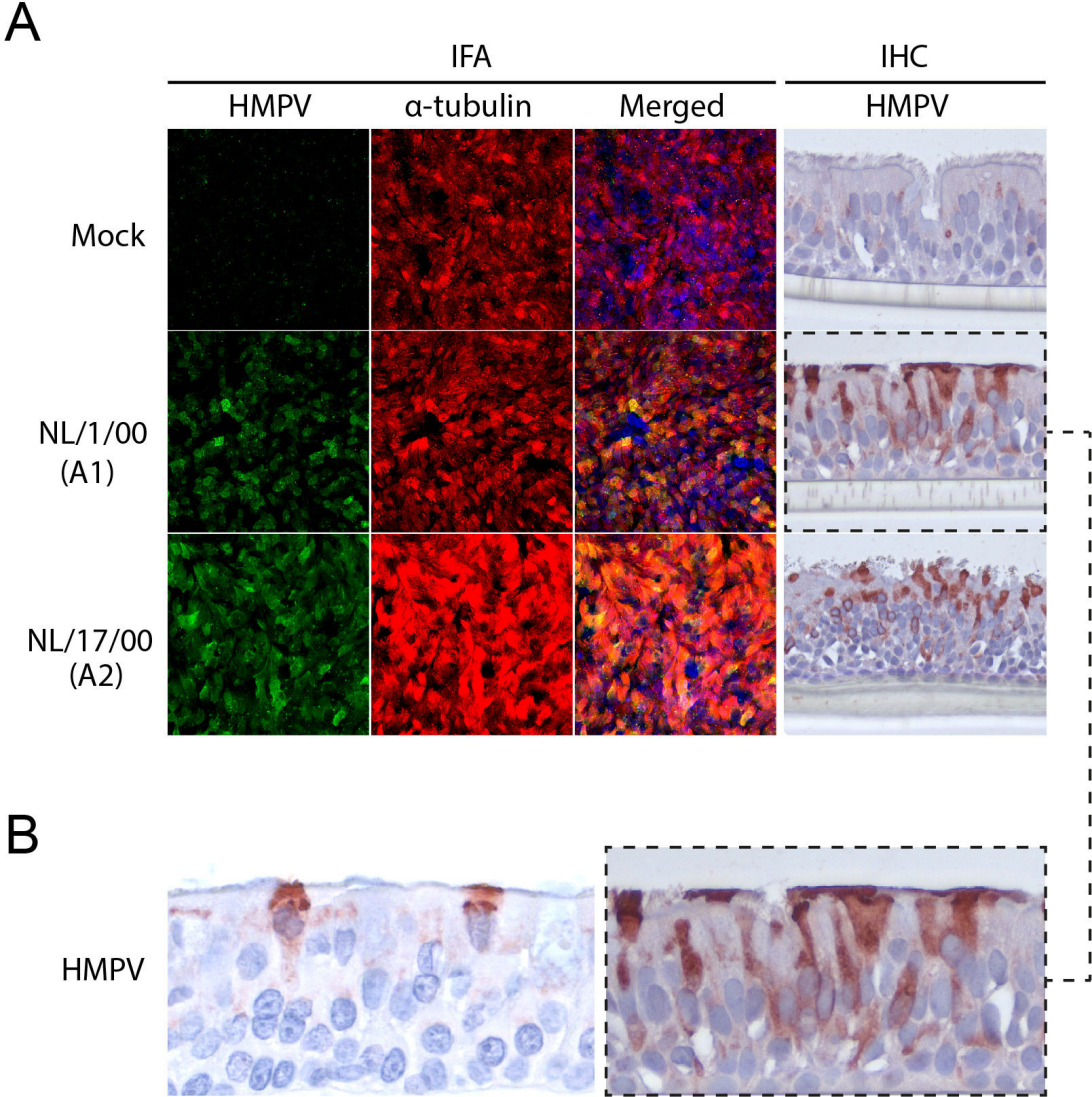
Immunostaining of bronchial cultures inoculated with prototype HMPV viruses. (**A**) IFA staining of bronchial cultures at 4 dpi with HMPV NL/1/00 (**A1**) and NL/17/00 (**A2**) at an MOI of 0.1, and IHC of the cross-section of the same bronchial culture. Immunofluorescence staining was performed using a polyclonal antibody against HMPV (green) and a monoclonal antibody against acetylated α-tubulin (cilia, red). Merged images are combined with Hoechst staining (nuclei, blue). (**B**) Immunohistochemistry staining for HMPV expression of a freshly cut and stained slide from a bronchial section of a cynomolgus macaque previously experimentally infected with HMPV NL/1/00 (14) (left) and bronchial cultures inoculated with HMPV NL/1/00 (right, highlighted with a dashed line from panel A). Samples for immunohistochemistry were fixed with formalin, embedded in paraffin, and immunostained for HMPV.

To examine the cellular tropism of HMPV, the bronchial cell cultures were examined by immunofluorescence assays (IFA) for the expression of HMPV antigens and a cilia marker, acetylated α-tubulin. At 4 days post-inoculation (dpi) with the four prototype viruses, expression of viral proteins was only observed in the cultures inoculated with genotype A viruses, NL/1/00 (A1) and NL/17/00 (A2), and the expression co-localized with the expression of the cilia marker. In accordance with the replication kinetics data (Fig. 1), no HMPV expression was observed in genotype B inoculated cultures (Fig. S1). Subsequently, cross-sections of the bronchial cultures were examined with immunohistochemistry (IHC) to confirm the cellular tropism (Fig. 3A). For comparison with *in vivo* studies, bronchial tissues of experimentally infected cynomolgus macaques, which were preserved from a previous study (14) were taken along. In both tissues, the expression of HMPV was restricted to ciliated cells, demonstrating that ciliated cells were the target of HMPV infection, without reaching the basal cells (Fig. 3B).

**Fig 4:**
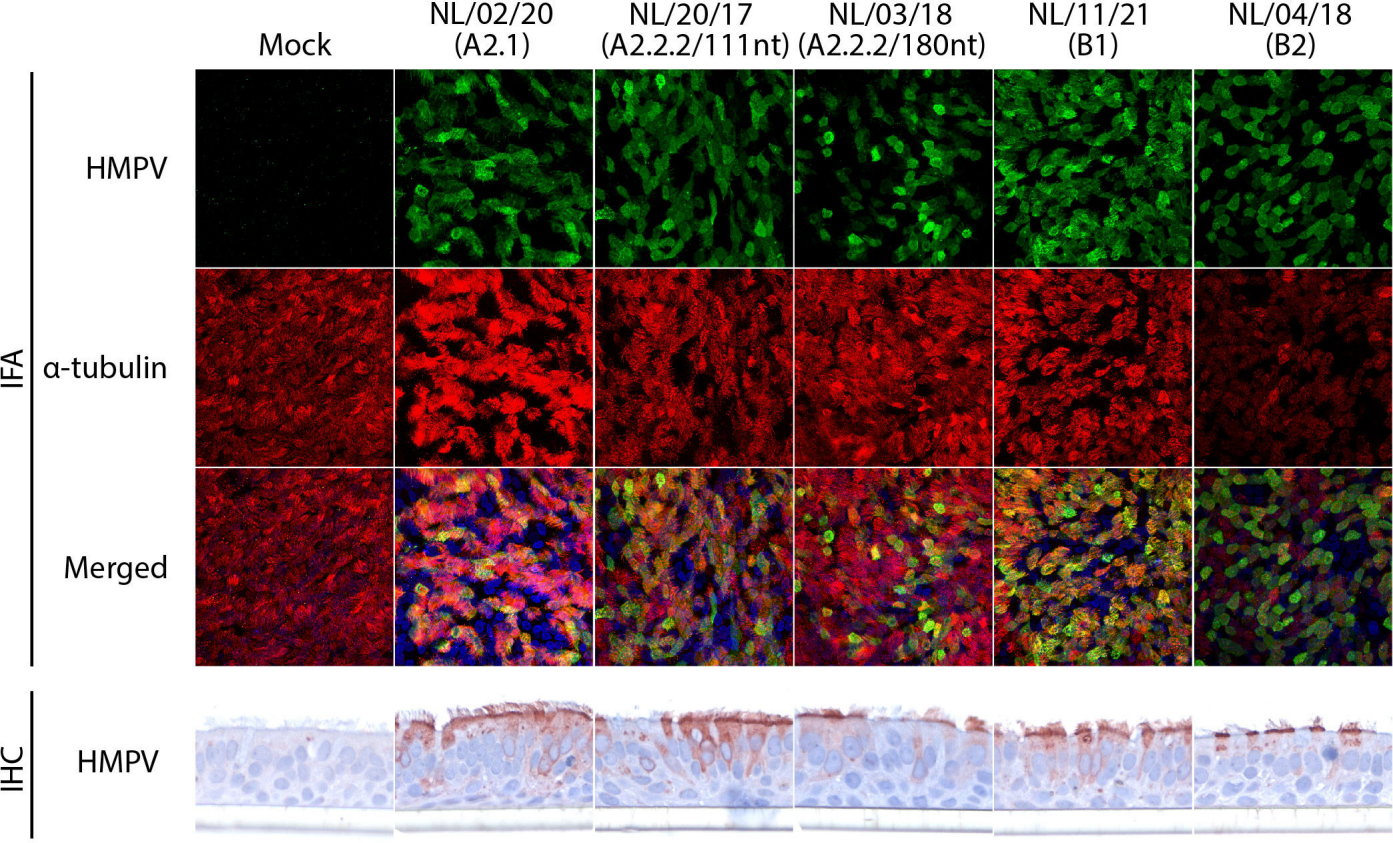
Immunostaining of bronchial cultures inoculated with recent HMPV isolates. (**A**) IFA staining of bronchial cultures collected at 5 dpi with HMPV NL/02/20 (A2.1), NL/20/17 (A2.2.2/180 nt), NL/03/18 (A2.2.2/111 nt), NL/11/21 (**B1**) and NL/04/18 (**B2**) at an MOI of 0.1, and IHC of the cross-section of the same bronchial culture. IFA and IHC staining were performed as detailed in Fig. 3.

To investigate the cellular tropism of the recent HMPV isolates, bronchial cultures inoculated with HMPV NL/02/20 (A2.1), NL/20/17 (A2.2.2/180 nt), NL/03/18 (A2.2.2/111nt), NL/11/21 (B1), and NL/04/18 (B2) were examined for viral protein expression at 5 dpi. In this case, viral protein expression was observed for all HMPV inoculated cultures and for all isolates’ protein expression co-localized with the expression of cilia markers in IFA (Fig. 4). IHC staining of cross-sections of the inoculated cultures confirmed the expression of viral proteins in ciliated cells (Fig. 4).

### HMPV damages the ciliary layer of organoid-derived bronchial cultures

**Fig 5:**
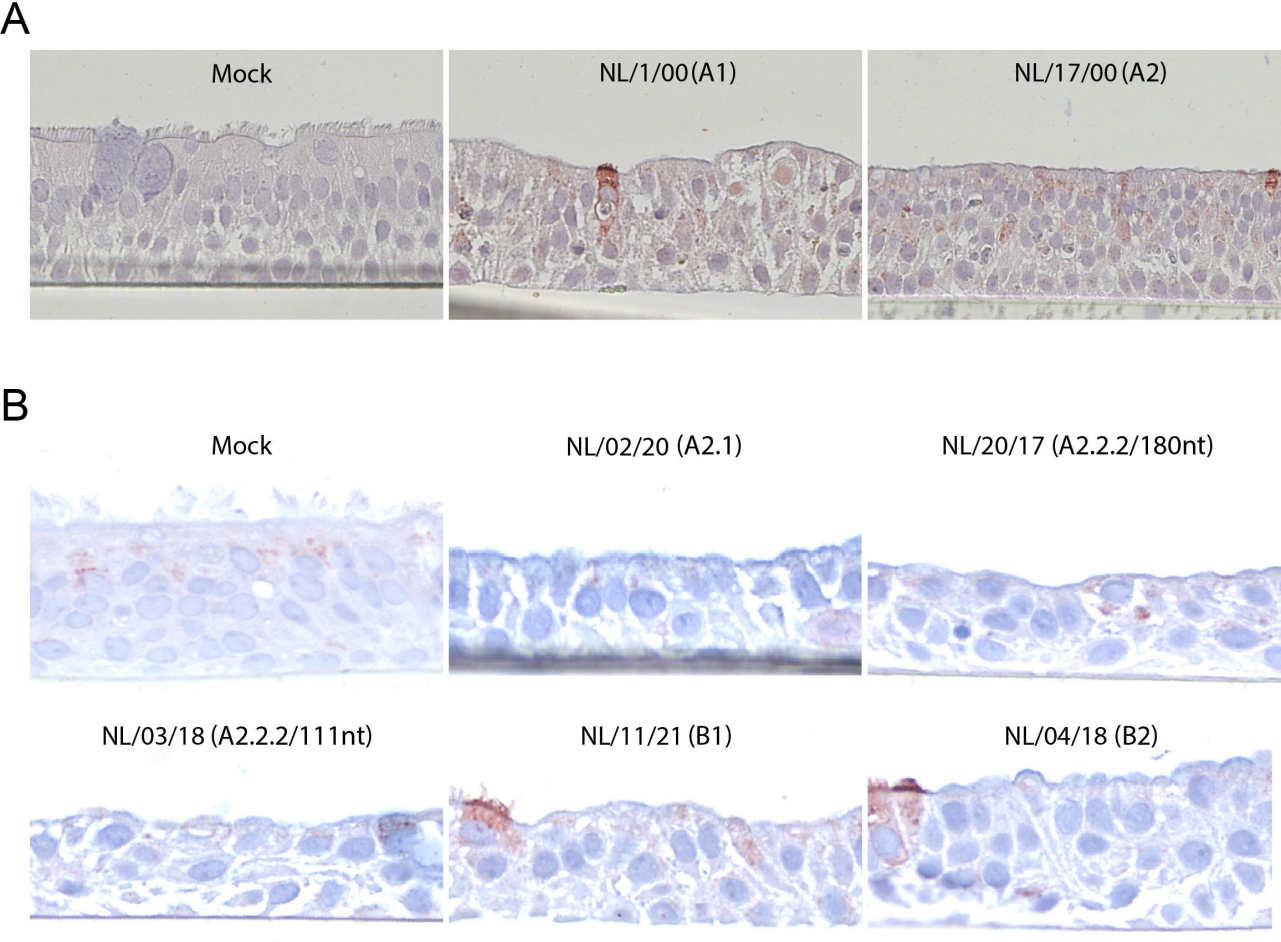
Immunohistochemistry staining of bronchial cultures at 8 days after inoculation with NL/1/00 (**A1**), NL/17/00 (**A2**), or 9 days after inoculation with NL/02/20 (A2.1), NL/20/17 (A2.2.2/180nt), NL/03/18 (A2.2.2/111nt), NL/11/21 (**B1**), and NL/04/18 (**B2**) at an MOI of 0.1. Mock-infected bronchial cultures were taken as control. Immunohistochemistry samples were fixed with formalin, embedded in paraffin, and immunostained with a polyclonal antibody against HMPV.

To assess whether HMPV infections induced cytopathic effects in the bronchial cells, cross-sections of inoculated bronchial cultures were examined by IHC at later stages post-infection. At 8 dpi, cilia damage was observed in cultures inoculated with HMPV genotype A viruses, resulting in only very few ciliated cells left that expressed HMPV antigens, correlating the loss of cilia with the decrease of viral antigen expression. Of note, the infection did not reach the basal cells (Fig. 5A).

To examine whether the recent isolates caused cellular damage, the bronchial cultures were evaluated 1 day later than the cultures inoculated with the earlier prototype viruses due to potentially slower replication kinetics of the recent isolates compared to the earlier prototype viruses. At 9 dpi with the recent HMPV isolates, all HMPV-inoculated bronchial cultures revealed damage to the ciliary layer (Fig. 5B). Here, only the cultures inoculated with HMPV NL/11/21 (B1) and NL/04/18 (B2) had remaining infected cells, in contrast to cultures inoculated with genotype A isolates, where all cilia were lost, again connecting the loss of cilia with the decrease in the expression of viral antigen. This observation correlated with a lower replication of the B lineage isolates than those from the A lineage. Again, HMPV infection did not reach the basal cells in the bronchial cultures.

### Cytokine response in organoid-derived bronchial epithelial cultures upon HMPV infection

To evaluate whether HMPV infection activated an innate immune response, apical washes were collected at designated time points, and expression levels of antiviral cytokines were determined by Legendplex assay. The cytokine responses were examined in bronchial cultures obtained from three different donors and at first evaluated for their response to the early prototype viruses of only genotype A, as the early prototype genotype B viruses did not replicate in this model. As a control, recombinant RSV A2 (rRSV A2) was taken along. RSV is the most closely related mammalian relative of HMPV, and induction of cytokine responses in this model has been reported before (23).

**Fig 6:**
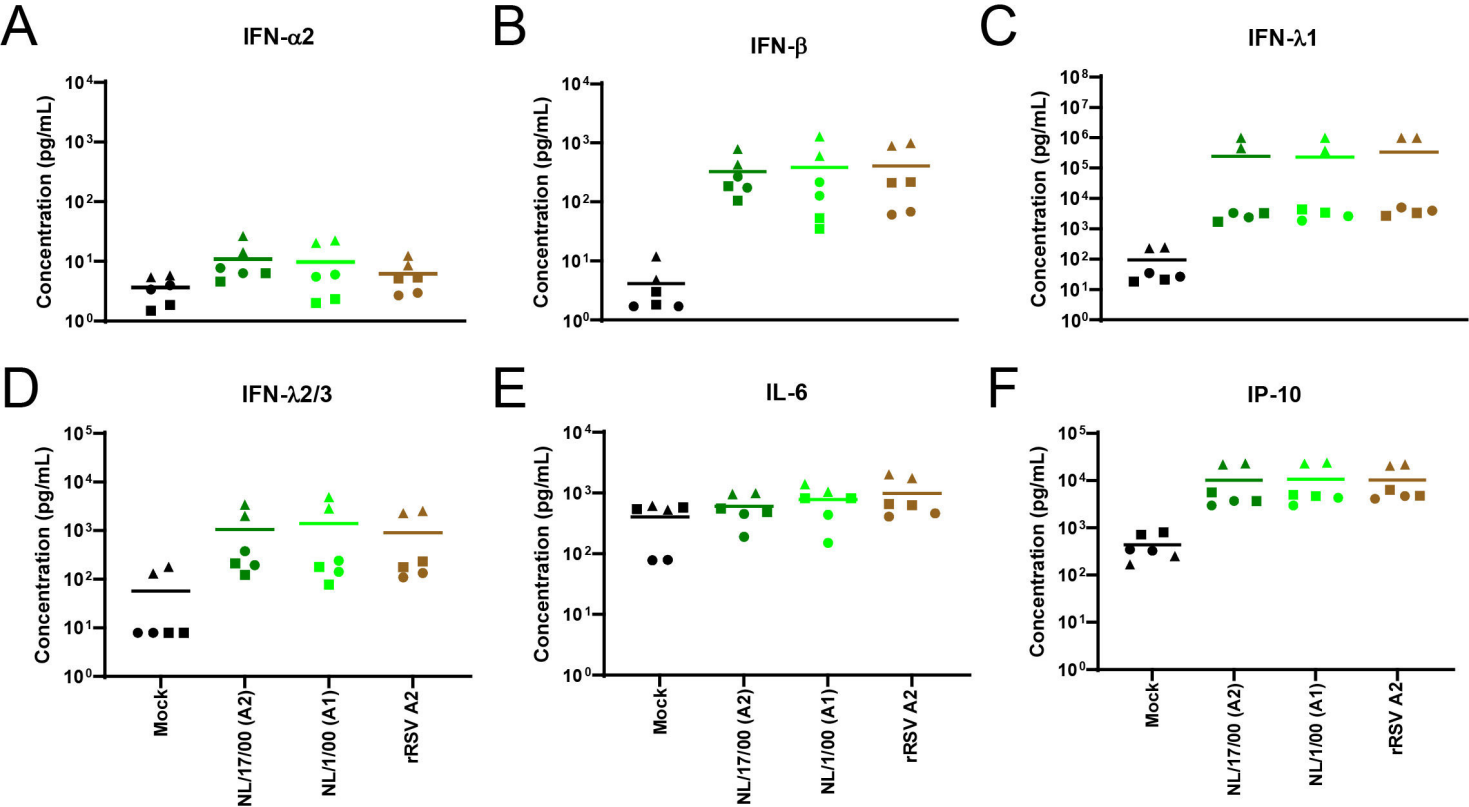
Virus-induced cytokine responses of bronchial cultures inoculated with prototype HMPV and rRSV A2. Inoculation performed at an MOI of 1 and at time point 96 hpi is shown. Samples were collected in duplo, and the experiment was conducted with three different donors. Cytokine expression levels were quantified with BD LEGENDplex Human Antivirus Response Panel, for which (A) IFN-α2, (B) IFN-β, (C) IFN-λ1, (D) IFN-λ2/3, (E) IL-6, and (F) IP-10 expression levels are shown. All data points are shown with the respective mean and symbols representing individual donors are consistent throughout the manuscript.

First, expression levels were determined at 2, 24, 48, 72, and 96 hpi, which showed the highest induction at 96 hpi (Fig. S2), and for clarity, only the results at 96 hpi are shown in Fig. 6. At 96 hpi with either HMPV or rRSV, only a very slight increase in IFN-α2 was observed in all three inoculated cultures upon inoculation with all viruses (Fig. 6A). Inoculation with HMPV induced similar levels of IFN-β expression as induced by rRSV A2 (Fig. 6B). Similarly, all three viruses induced similar expression of IFN lambda-1 (IFN-λ1) and IFN-λ2/3, where expression levels in one donor were higher than in the other two donors (Fig. 6C and D). In addition, inoculation with all three viruses led to a minor increase in expression levels of the pro-inflammatory cytokine interleukin-6 (IL-6), but to higher expression levels of IP-10 compared to mock-infected cultures, with similar levels induced by rRSV A2 and by the two HMPV prototype virus (Fig. 6E and F). Overall, the trend in cytokine expression observed for the three donors was similar, although one of the three donors presented higher cytokine background and virus-induced expression levels for most of the cytokines.

**Fig 7:**
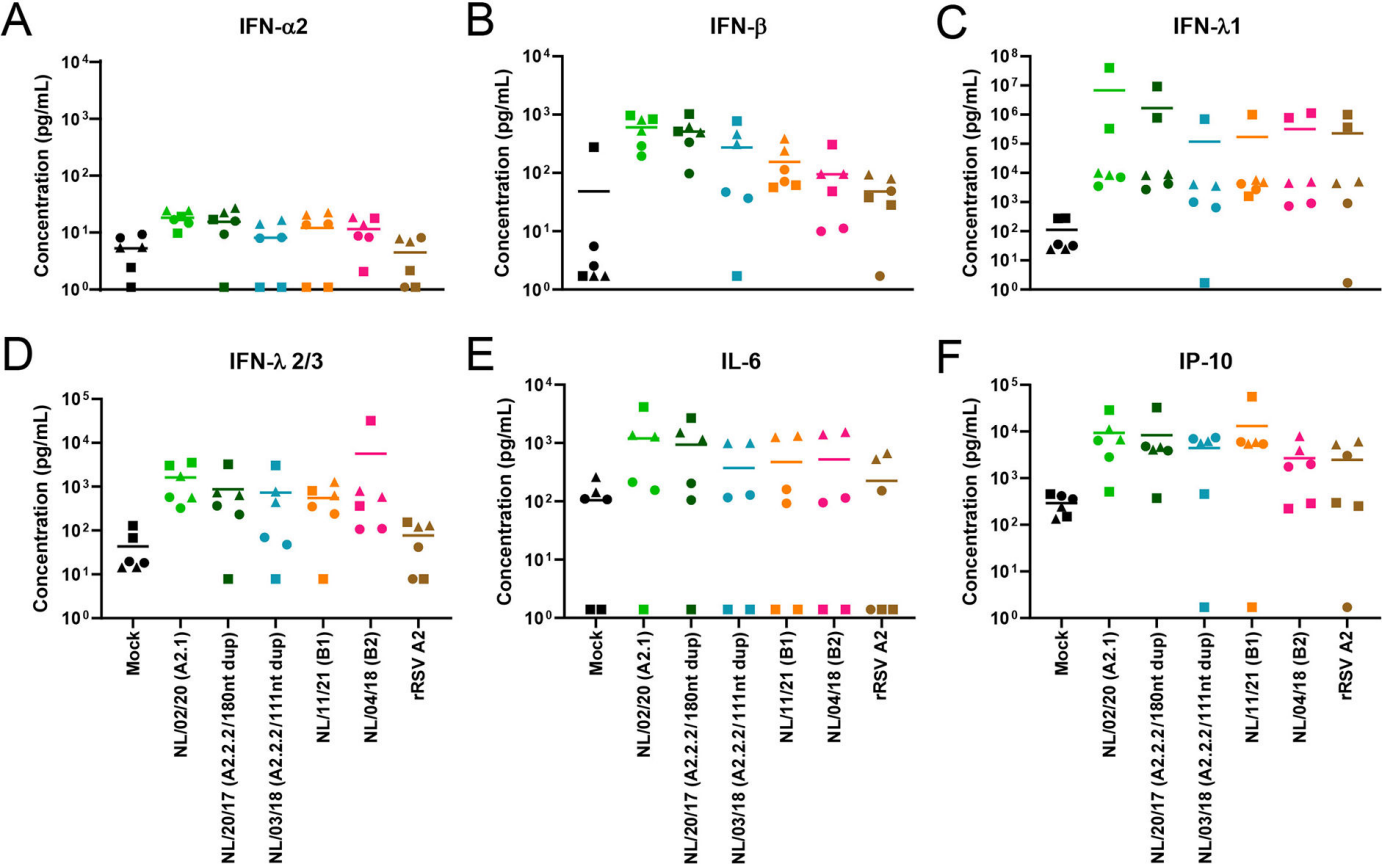
Innate cytokine response induced upon infection of bronchial cultures with recent HMPV isolates and rRSV A2. Bronchial cultures were inoculated at an MOI of 1 and at time point 72 hpi is shown. Samples were collected in duplo, and the experiment was conducted with three different donors. Cytokine expression levels were quantified with BD LEGENDplex Human Antivirus Response Panel, for which (A) IFN-α2, (B) IFN-β, (C) IFN-λ1, (D) IFN-λ2/3, (E) IL-6, and (F) IP-10 are shown. All data points are shown with the respective mean and symbols representing individual donors are consistent throughout the manuscript.

Inoculation of the bronchial cultures from three donors with the recent virus isolates resulted in a similar pattern of cytokine responses, although more variation between donors was observed among donors. Here, the responses peaked at 72 hpi, instead of 96 hpi as observed upon inoculation with the prototype viruses (Fig. S3). The IFN-α2, IFN-β, IFN-λ1, and IFN-λ2/3 responses to these recent isolates were also slightly higher than toward the older prototype viruses and slightly higher than to rRSV A2. Among the recent isolates, the highest responses were observed for HMPV A2.1 and A2.2.2 with a 180 nt duplication, in accordance with a more efficient replication observed for these two isolates in the bronchial cultures (Fig. 7A through D). Inoculation with all five recent isolates induced an increase in the expression of the proinflammatory cytokines IL-6 and IP-10 to slightly higher levels than upon inoculation with the earlier prototype viruses or rRSV A2 (Fig. 7E and F). However, this was only observed for two of the donors, as the third donor induced IL-6 expression levels below the limit of detection and IP-10 detection levels below the limit of detection in one of the two duplo measurements. These observations highlighted the value of including multiple donors to study innate immune responses.

No increase in expression levels of IL-1β, IL-12p70, IL-8, IL-10, IFN-γ, TNF-α, or GM-CSF was observed in inoculated bronchial cultures with either set of the HMPV isolates or with rRSV A2 compared to the mock (Fig. S4 and S5).

## DISCUSSION

In this study, an organoid-derived bronchial culture was established as a potentially physiologically relevant model for studying HMPV infection. To assess the permissiveness of the model to HMPV infection, bronchial cultures were initially inoculated with early prototype viruses for the four genotypes. Here, replication was only observed for the prototype HMPV viruses of the A lineage but not for those of the B lineage. Subsequent evaluation of recently isolated viruses from all HMPV genotypes revealed that all these isolates replicated efficiently in this model, suggesting a potential adaptation for the older HMPV B prototype viruses to long-term cell propagation. The similar susceptibility for HMPV infection of the bronchial cultures obtained from three different donors indicated the robustness of this model to study HMPV infections.

Due to the lower endpoint titers obtained for the recent HMPV isolates compared to the older prototype viruses, the bronchial cultures inoculated with the recent isolates were sampled for an additional day. However, in immunostaining of the inoculated cultures, limited differences were detected between the older prototype genotype A viruses and the recent isolates. The lower endpoint titers reached by the recent HMPV isolates could be explained by a more advantageous infection of Vero-118 cells during titration of the older prototype HMPV viruses being adapted to these cells rather than a more prolific infection of the bronchial cultures.

A limited number of studies have used differentiated human airway epithelial cells to study HMPV infection. Recent studies assessing HMPV replication in two- and three-dimensional primary epithelial cell models had limited success, in contrast to similar studies with RSV (17, 18). In previous studies with human primary bronchial epithelial cells cultured at ALI, inoculation with NL/1/99 (B1) at a high MOI resulted in limited infection (26), which was also observed in our bronchial model. Similarly, infection of reconstituted human nasal airway epithelium (HAE) showed limited replication of a different genotype B virus, CAN97-82, up to 5 days post-inoculation (21). Another study showed that rHMPV CAN97-83 (A2) replicated poorly in differentiated HAE MucilAir epithelium, in contrast to RSV (18). Finally, a study using HMPV rCAN98-75 (B2) and rC85473 (A1) suggested that rHMPV harbored isolate-dependent replicative properties in the HAE MucilAir epithelium. That study suggested that, while both rHMPV A1 and B2 did spread in the HAE cultures, the glycoprotein G was critical for HMPV B2 infection in contrast to infection with HMPV A1 (19). The aforementioned differences highlight the importance of including viruses with a controlled passage history in these types of studies, instead of extensively passaged HMPV strains. Moreover, in agreement with Kinder et al. (18), the isolate-to-isolate variation occurring among genotypes is understudied and should also be considered when using primary lung cultures.

Here, we demonstrated successful infection of the bronchial cultures with several HMPV isolates of all genotypes and confirmed that ciliated cells are a prime target for HMPV infection, with subsequent deciliation, in accordance with previous studies in cynomolgus macaques and cotton rats, where multi-focal cilia loss was observed in airway tissues (14, 15).

Despite the lack of immune cells in these cultures, which are present in an *in vivo* setting, airway ALI models are a valuable model to study innate immune responses (23). The cytokine responses upon RSV inoculation in our bronchial culture model corroborated the results described in a previous study with the same bronchial cultures (23). RSV is known to evade innate immune responses by means of its IFN antagonist proteins NS1 and NS2 (reviewed in reference 27). In this study, the cytokine responses elicited upon HMPV infection were comparable to those upon rRSV inoculation and included moderate secretion of type I and III IFNs, as well as pro-inflammatory cytokines IL-6 and IP-10. These virus-induced responses were insufficient to prevent HMPV replication, confirming previous reports on HMPV’s abilities to subvert innate immune responses (28, 29). However, more studies are needed to identify the mechanism by which HMPV subverts the IFN response in relevant models, including the donor variability when studying innate immune responses, as pinpointed by this study.

Altogether, the observed tropism, induced cytopathic effects, and cytokine responses in this model further affirm its suitability as a robust model and valuable substitute to study HMPV infection in a manner that closely resembles *in vivo* observations. The use of recent HMPV isolates with a limited passage history allowed for validation of the model for a broad range of HMPV isolates from various lineages.

## MATERIALS AND METHODS

### Cells and viruses

Vero-118 cells were cultured as described previously (29) and were used to generate stocks of wild-type HMPV NL/1/00 (A1), NL/17/00 (A2), NL/1/99 (B1), NL/1/94 (B2), NL/02/20 (A2.1), NL/20/17 (A2.2.2/180 nt), NL/03/18 (A2.2.2/111 nt), NL/11/21 (B1), and NL/04/18 (B2). Recent HMPVs were isolated from samples obtained from hospitalized patients in the Netherlands (9). Prototype HMPVs were long-term passaged in Vero-118 cells, with potential cell culture adaptation, whereas the recent HMPV isolates were passaged at a low MOI until passage 3. The rRSV (A2) stock was passaged at low MOI until passage 2, after recovery from cDNA in a similar manner as described previously (30). The vector containing the complete RSV A2 cDNA clone T306 was a kind gift from Professor Dr. A. G. P. Oomens (Department of Veterinary Pathology, Oklahoma State University). Infectious virus titers were determined by endpoint dilution in Vero-118 cells as described previously (29).

### Bronchial airway organoid culture and differentiation

The culture of human bronchial airway organoids was achieved as described before (25, 31). Isolation of stem cells was performed from adult lung tumor-free tissue (Medical Ethical Committee of the Erasmus MC Rotterdam, METC 2012-512). Stem cells were grown into undifferentiated airway organoids (AO), cultured in Basement Membrane Extract type 2 (3536-005-02, Bio-Techne) droplets and AO medium as defined before (31). Differentiation from single cells to bronchial cultures in transwell systems in ALI was performed using PneumaCult Medium (StemCell Technologies).

### RNA isolation and qRT-PCR

RNA was isolated from 60 µL of apical wash in 90 µL MagNA Pure External Lysis Buffer (cat. no. 06374913001, Roche). Lysates were stored at −20°C until further RNA extraction. In brief, lysates were incubated for 15 min with Agencourt AMPure XP beads (cat. no. 10453438, Beckman Coulter). After two washes with 70% ethanol on a DynaMag-96 magnet (Invitrogen), RNA was eluted in 50 µL Bidest water. Next, 5 µL of eluted RNA was used for the assessment of HMPV N expression by quantitative TaqMan real-time PCR in a similar approach as described before (32).

### Immunofluorescence microscopy

Transwell inserts were washed twice with PBS + CaMg (Lonza) and fixed with 4% paraformaldehyde in PBS at room temperature (RT). Subsequently, samples were washed once with PBS and were stored at 4°C until staining. Permeabilization was performed with PBS 0.1% Triton X-100 per well for 10 min at RT. Cells were washed once with PBS and blocked with PBS 10% normal goat serum (NGS) for 1 hour. For single staining, coverslips were incubated in droplets with 50 µL in-house generated polyclonal anti-HMPV guinea pig antiserum (dilution 1:200), prepared as described previously (14). After washing, cells were incubated in droplets of 2% NGS/2% BSA with FITC rabbit anti-guinea pig immunoglobulins (DAKO, 1:100) and directly labeled AF647 acetylated a-tubulin (Santa Cruz Biotechnology, 1:100). After washing, nuclei were stained with Hoechst 20 µM (33342, Life Technologies) in PBS. Finally, coverslips were mounted using ProLong Gold Antifade Mountant solution (Invitrogen). Images were taken on a LSM700 confocal microscope, using ZEN software (Zeiss). The remaining analysis was performed using Image J software.

### Immunohistochemistry

Formalin-fixed, paraffin-embedded sections of bronchial cultures were mounted on coated slides (Klinipath). Slides of bronchial sections of cynomolgus macaques infected with HMPV NL/1/00 from Kuiken et al. (14) were stained in parallel. Sections were deparaffinized, rehydrated, and boiled for 15 min in citric acid buffer (pH 6.0) using a microwave oven. Endogenous peroxidase was blocked using H_2_O_2_, and slides were blocked in 10% normal rabbit serum. Sections were subsequently washed with PBS containing 0.05% Tween 20 (Fluka, Chemie AG, Buchs, Switzerland) and incubated with an in-house generated polyclonal guinea pig antiserum (dilution 1:200) to hMPV. After washing, sections were incubated with horseradish peroxidase (HRP)-labeled rabbit anti-guinea pig Ig (DAKO, 1:200) for 1 hour at room temperature. HRP activity was revealed by incubating the sections in 3-amino-9-ethylcarbazole (Sigma Chemical Co.) solution for 10 min, resulting in red staining. Sections were counterstained with hematoxylin and were examined by light microscopy, as previously described (14).

### Cytokine measurement

Apical washes were performed with PBS + CaMg (Lonza) at designated time points. Cytokine expression levels were measured using the Human Antivirus Response Panel (13-plex) kit (LEGENDplex; BioLegend) according to the manufacturer’s instructions. Analysis was performed using the LEGENDplex analysis software Qognit (version 2023-02-15). Cytokine expression levels were quantified based on a freshly prepared standard. The cytokine values below the minimum detectable concentration (MDC) were set to the suggested MDC by the LEGENDplex Data Analysis Software.

## SUPPLEMENTAL MATERIAL

Supplemental figures msphere.00743-23-s0001.docx Figures S1 to S5.

ASM does not own the copyrights to Supplemental Material that may be linked to, or accessed through, an article. The authors have granted ASM a non-exclusive, world-wide license to publish the Supplemental Material files. Please contact the corresponding author directly for reuse.
