# Supplementary material for: Human metapneumovirus infection of organoid-derived human bronchial epithelium represents cell tropism and cytopathology as observed in in vivo models
Source: mSphere. 2024 Jan 24;9(2):e00743-23. doi: 10.1128/msphere.00743-23 (PMC10900881; doi:10.1128/msphere.00743-23)
Supplement: Supplemental figures — Figures S1 to S5. [file msphere.00743-23-s0001.docx]

**Supplementary figure 1**

**
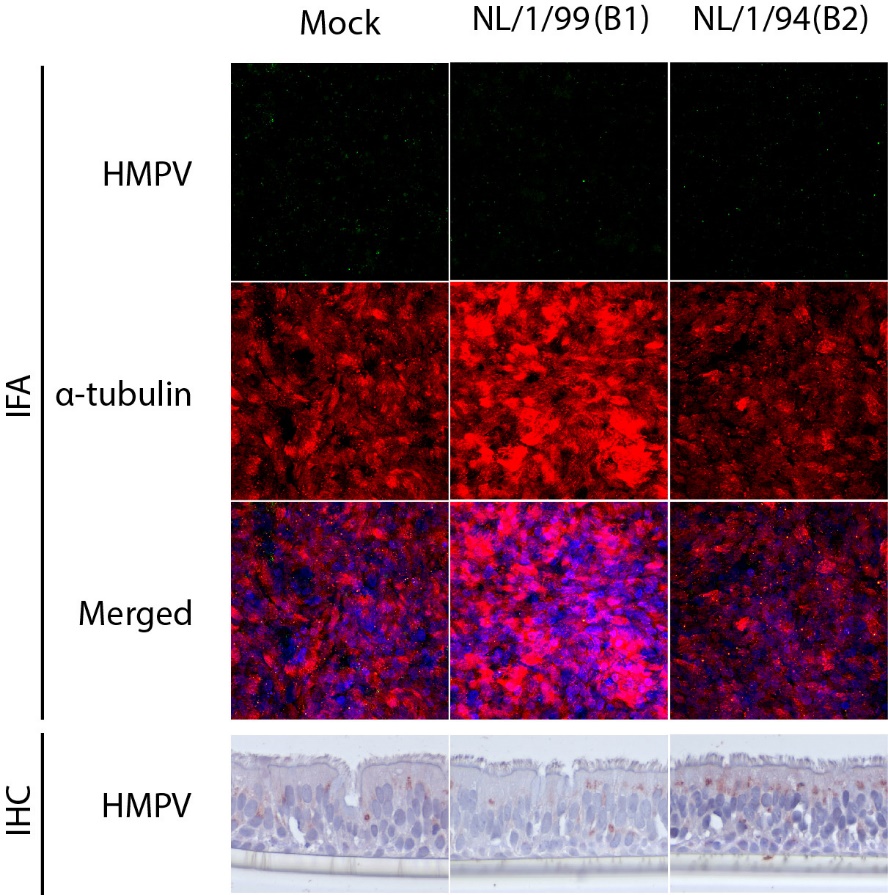
**


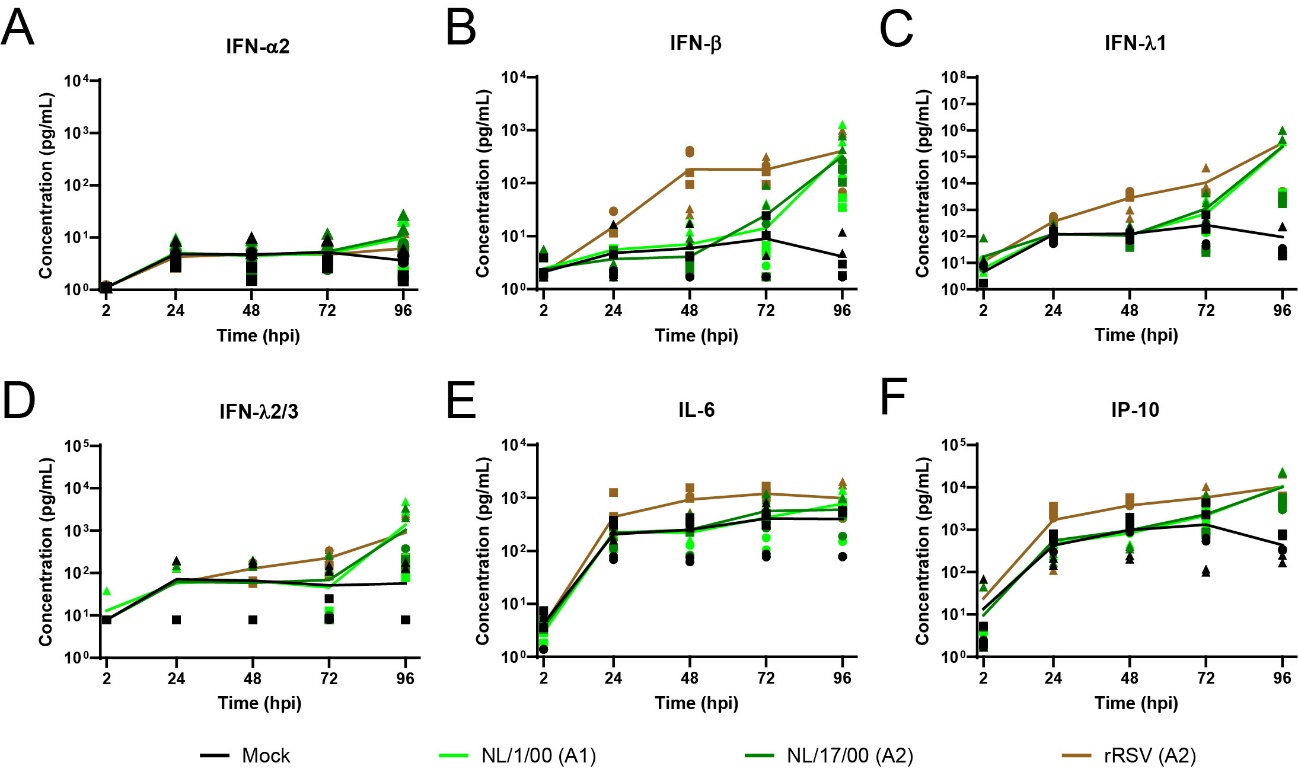
**Supplementary figure 2**

**
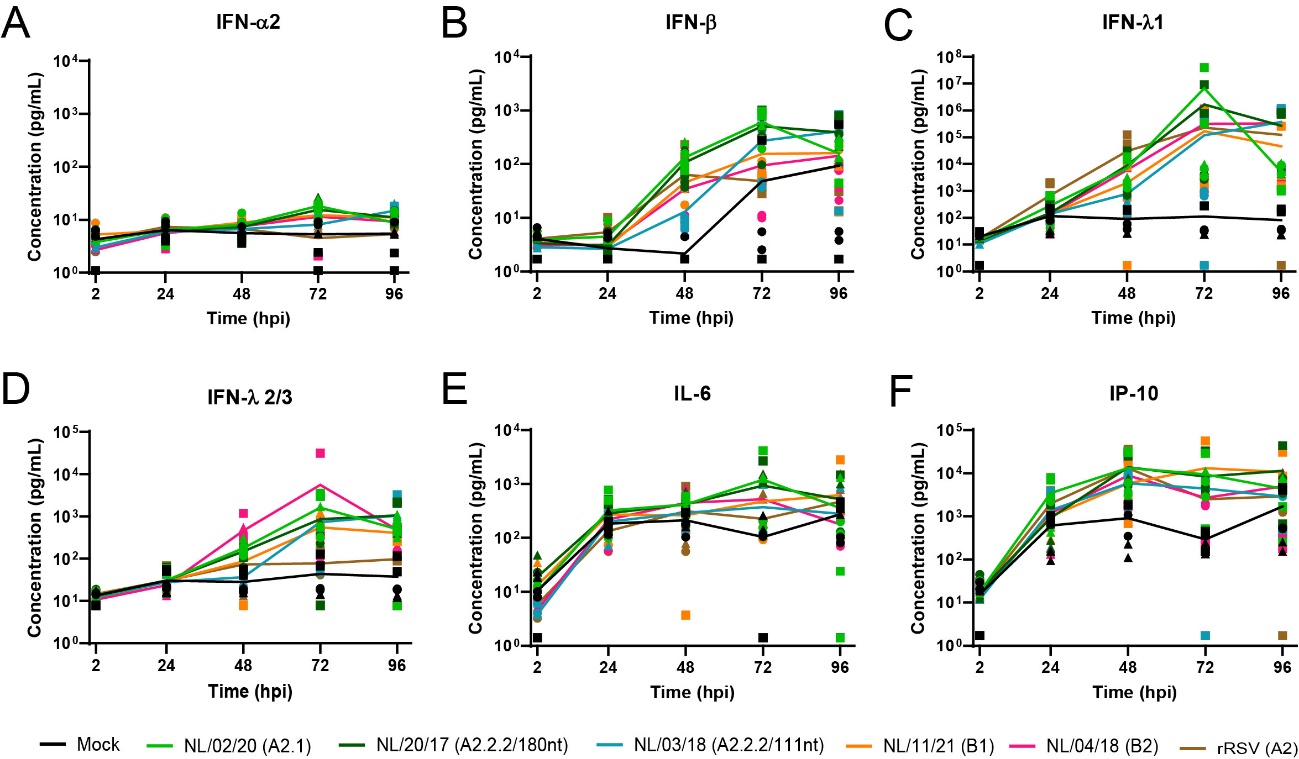
Supplementary figure 3**

**
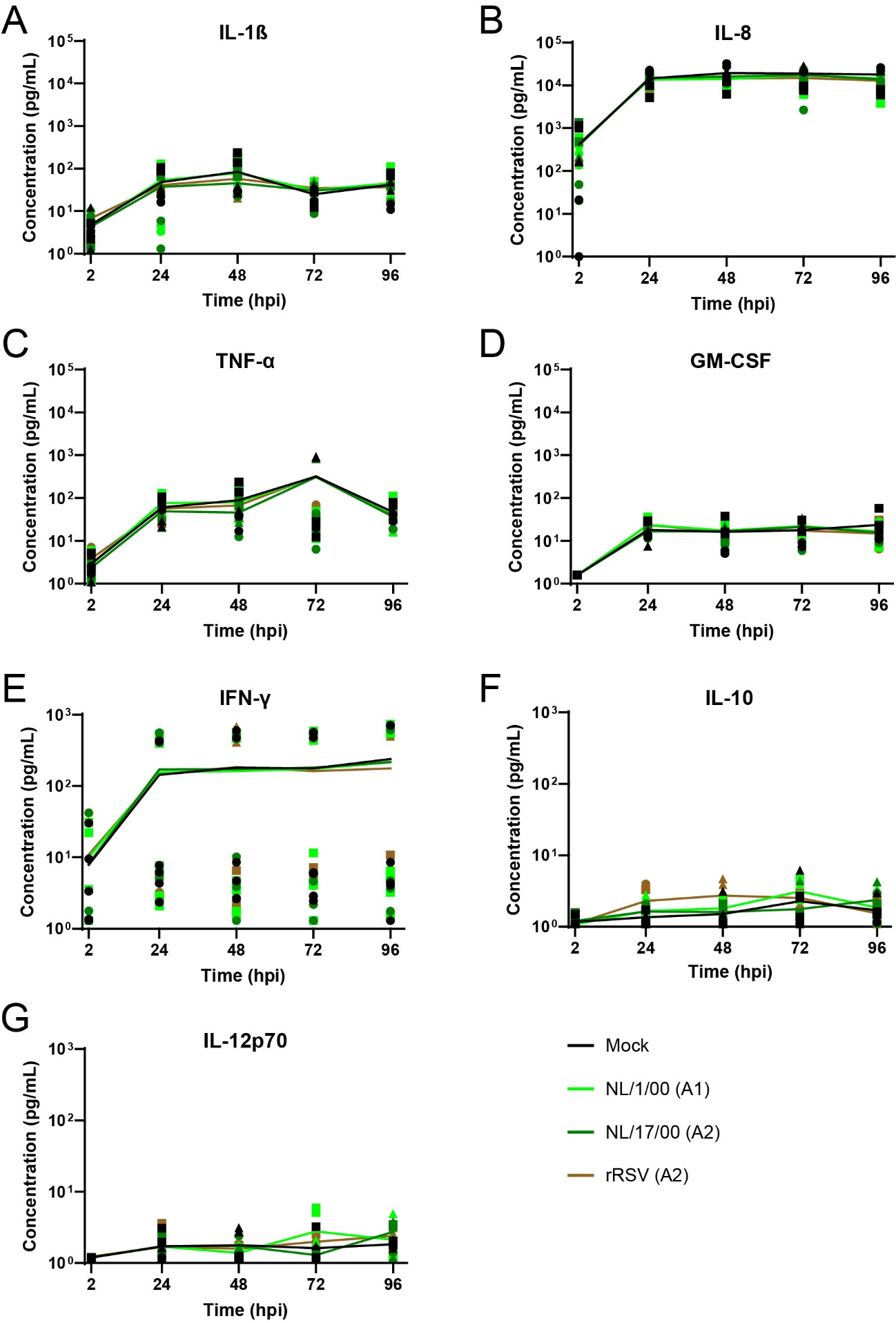
Supplementary figure 4**

**Supplementary figure 5**

**
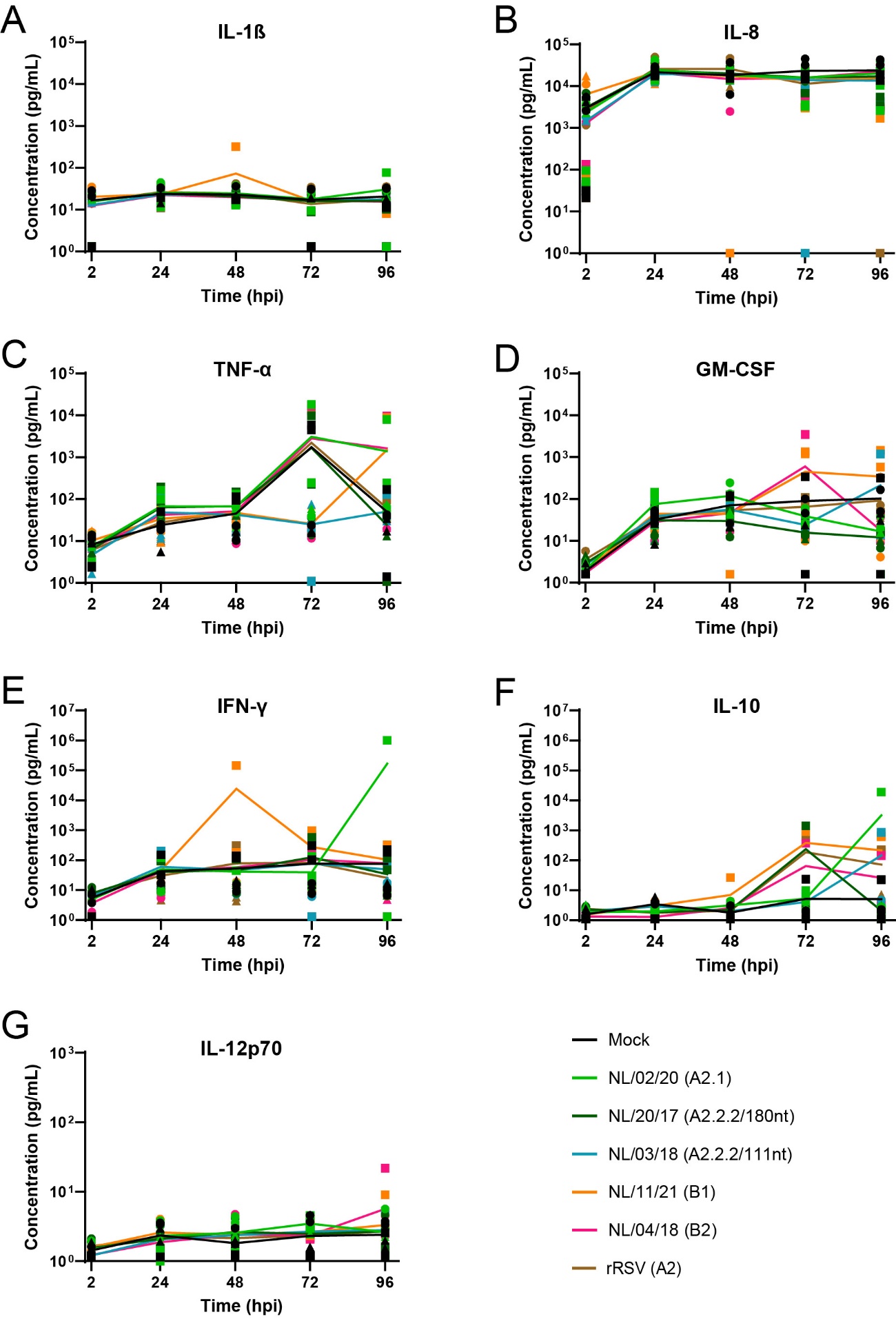
**

**Supplementary figure legends**

**Supplementary figure 1.** Immunostainings of bronchial cultures inoculated with prototype HMPV B viruses. Mock-infected bronchial cultures from figure 3 are displayed for clarity. Immunofluorescence assay (IFA) staining of bronchial cultures at 4 dpi with HMPV NL/1/99 (B1) and NL/1/94 (B2) at an MOI of 0.1, and immunohistochemistry (IHC) of the cross-section of the same bronchial culture. Immunofluorescence staining was performed using a polyclonal antibody against HMPV (green) and a monoclonal antibody against acetylated α-tubulin (cilia, red). Merged images are combined with Hoechst staining (nuclei, blue).

**Supplementary figure 2.** Virus induced cytokine responses of bronchial cultures inoculated with prototype HMPV and rRSV A2. Inoculation was performed at an MOI of 1 and apical washes were sampled at 2, 24, 48, 72 and 96hpi. Samples were collected in duplo and experiment was conducted with three different donors. Cytokine expression levels were quantified with BD LEGENDplex Human Antivirus Response Panel, for which IFN-α2, IFN-β, IFN-λ1, IFN-λ2/3, IL-6 and IP-10 expression levels are shown. All data points are shown with the respective mean and symbols representing individual donors are consistent throughout the manuscript.

**Supplementary figure 3.** Innate cytokine response induced upon infection of bronchial cultures with recent HMPV isolates and rRSV A2. Bronchial cultures were inoculated at an MOI of 1 and apical washes were sampled at 2, 24, 48, 72 and 96hpi. Samples were collected in duplo and the experiment was conducted with three different donors. Cytokine expression levels were quantified with BD LEGENDplex Human Antivirus Response Panel, for which IFN-α2, IFN-β, IFN-λ1, IFN-λ2/3, IL-6 and IP-10 are shown. All data points are shown with the respective mean and symbols representing individual donors are consistent throughout the manuscript.

**Supplementary figure 4.** Virus induced cytokine responses of bronchial cultures inoculated with prototype A HMPV and rRSV A2. Inoculation was performed at an MOI of 1 and apical washes were sampled at 2, 24, 48, 72 and 96hpi. Samples were collected in duplo and experiment was conducted with three different donors. Cytokine expression levels were quantified with BD LEGENDplex Human Antivirus Response Panel, for which IL-1β, IL-8, TNF-α, GM-CSF, IFN-γ, IL-10 and IL-12p70 expression levels are shown. All data points are shown with the respective mean and symbols representing individual donors are consistent throughout the manuscript.

**Supplementary figure 5.** Innate cytokine response induced upon infection of bronchial cultures with recent HMPV isolates and rRSV A2. Bronchial cultures were inoculated at an MOI of 1 and apical washes were sampled at 2, 24, 48, 72 and 96hpi. Samples were collected in duplo and the experiment was conducted with three different donors. Cytokine expression levels were quantified with BD LEGENDplex Human Antivirus Response Panel, for which IL-1β, IL-8, TNF-α, GM-CSF, IFN-γ, IL-10 and IL-12p70 are shown. All data points are shown with the respective mean and symbols representing individual donors are consistent throughout the manuscript.
